# miRNAs and target genes in the blood as biomarkers for the early diagnosis of Parkinson’s disease

**DOI:** 10.1186/s12918-019-0680-4

**Published:** 2019-01-21

**Authors:** Xiaoting Liu, Jinhu Chen, Tianyuan Guan, Hui Yao, Wenpei Zhang, Zhenlong Guan, Yanqin Wang

**Affiliations:** 1 Department of Physiology, College of Life Sciences, Hebei Normal University, Shijiazhuang, China; 2 Department of Endocrinology, Hebei General Hospital, Shijiazhuang, China; 3 Department of Neurology, Hebei General Hospital, Shijiazhuang, China

**Keywords:** Parkinson’s disease, Functional enrichment analysis, Protein–protein interaction, miRNA-mRNA pairs

## Abstract

**Background:**

Parkinson’s disease (PD) is the second most common neurodegenerative disease, and it is a multifactorial disease with no definite diagnostic index. The aim of this study is to construct a molecular network to find molecules that play important roles in the progression of PD with the goal of using them diagnostically and for early intervention.

**Results:**

We downloaded two gene expression profiles (GSE54536 and GSE100054) from the Expression Omnibus (GEO) database to analyze possible markers. The Genes were analyzed with GEO2R. There were 1790 and 967 differentially expressed genes (DEGs) in GSE54536 and GSE100054 respectively. A total of 125 genes co-exist in the DEGs of the two data sets. KEGG pathway analysis showed that 125 DEGs were enriched in Aldosterone synthesis and secretion, Gap junctions, Platelet activation, Rap1 signaling pathway, and Estrogen signaling pathway. There were 20 hub genes among 125 DEGs analyzed by PPI that involved in Platelet activation, Inflammatory response, Innate immune response, B cell receptor signaling, Stimulatory C-type lectin receptor signaling, Lipopolysaccharide response, Leukocyte migration, and Regulation of cell proliferation. Additionally, 42 differences in miRNAs were found in GSE100054. We constructed a miRNA-mRNA regulatory network depicting interactions between the predicted genes and the 125 DEGs. 34 miRNA-mRNA pairs were obtained. We found GNAQ and TMTC2 were the most important mRNAs in the network analyzed by Cytoscape APP centiscape, and their degrees in centiscape2.2 were all 10. has-miR-142 was the most important miRNA (the highest degree is 4 in centiscape2.2), which forms miRNA-mRNA pairs with GNAQ, TMTC2, BEND2, and KYNU.

**Conclusions:**

This study provides data of potential biomarkers and therapeutic targets for PD diagnosis and treatment. Among them, hsa-miR-142 is a critical miRNA in the PD network, and may be involved in PD progression by regulating GNAQ, TMTC2, BEND2, and KYNU.

## Background

Parkinson’s disease (PD) is the second most common neurodegenerative disease behind Alzheimer’s disease. It has a 1% incidence in people 65 years of age [1], and 4% in those 80 years of age [2]. Early stage PD patients showed only non-motor symptoms (NMS), such as constipation, olfactory dysfunction, depression, and sleep disorders [3]. Clinical motor symptoms included bradykinesia, stiffness, tremor and postural instability, and asymmetric seizures. Other motor dysfunctions included gait and posture changes (manifesting as panic gait or walking forward chaotically with rapid bending), speech and swallowing difficulties, and masked expressions [4]. When people displayed clinical symptoms, there was an associated loss of at least 50% of dopaminergic neurons in the substantia nigra (SN), and an 80% decrease in striatal DA content. Early identification of PD molecular biomarkers is critical for initiating timely treatment prior to the onset of motor symptoms. This is especially true given that 90% of rapid eye movement sleep behavior disorder (RBD) patients develop PD in most cases, Do non-motor symptoms need change to NMS? precede motor symptoms [5]. However, these Do non-motor symptoms need change to NMS? are easily overlooked. Therefore, the early recognition and treatment of Do non-motor symptoms need change to NMS? is not only one of the most important and difficult issues in the current diagnosis and treatment of PD, but also directly affects the overall therapeutic effect on PD patients. Currently, the pathogenesis of PD is not clear. PD is caused by a variety of factors, including age, genetics, oxidative stress, calcium overload, mitochondrial dysfunction, and environmental factors. Previous studies have focused on individual pathogenic factors, but PD is not caused by a single factor, and there is cross-talk among many of the causal elements. These factors form a regulatory network, and we should re-examine Do Parkinson's disease need change to PD? adopting a network perspective to better comprehend the complex interplay of causes. Due to the scarcity of brain tissue, it is necessary to establish a database of clinical data of PD patients and bioinformatic libraries to explore incidence, related risk factors, biomarkers, occurrence, and development.

MicroRNAs (miRNAs) are a class of noncoding RNA molecules that are single-stranded and 19–25 nucleotides long. They are involved in proliferation, apoptosis, differentiation, metabolism, and development of cells by degrading or mediating translation by complementarity to the 3’UTR of target RNAs [6–8]. Since miRNAs are a key mechanism for controlling gene expression, regulatory miRNAs and their target genes can serve as biomarkers for the early diagnosis of PD. miRNAs and their derivatives have great potential in the diagnosis and treatment of diseases [8–11].

With the development of high-throughput sequencing technology, existing data can be used for screening, comparing, and analyzing information that otherwise would not be available from data collected from only a few individuals. Data from GEO datasets regarding the expression profiling of peripheral blood were used to screen normal controls and PD patients. Determining differentially expressed genes(DEGs) and signaling pathways helped us identify key molecules and screening targets for the early diagnosis and development of PD. Here, we used two datasets, GSE54536 and GSE100054, of peripheral blood RNA and miRNA from PD patients and determined differences in mRNAs between controls and PD samples. The DEGs were introduced into the DAVID website, the differentially expressed genes were annotated, and the biological processes, cell components, molecular functions, and signaling pathways involved in DEGs of PD patients were analyzed. Differentially expressed microRNAs (DEMs) were introduced into Targetscan website to predict the miRNA target genes. The analyzed mRNAs and miRNAs were jointly analyzed to screen for miRNA-mRNA pairs. To explore the pathogenesis of PD from the perspective of transcriptomes, these miRNA-mRNA pairs may be potential targets for future research in the prevention and treatment of PD.

## Methodology

### Data collection

**Fig. 1:**
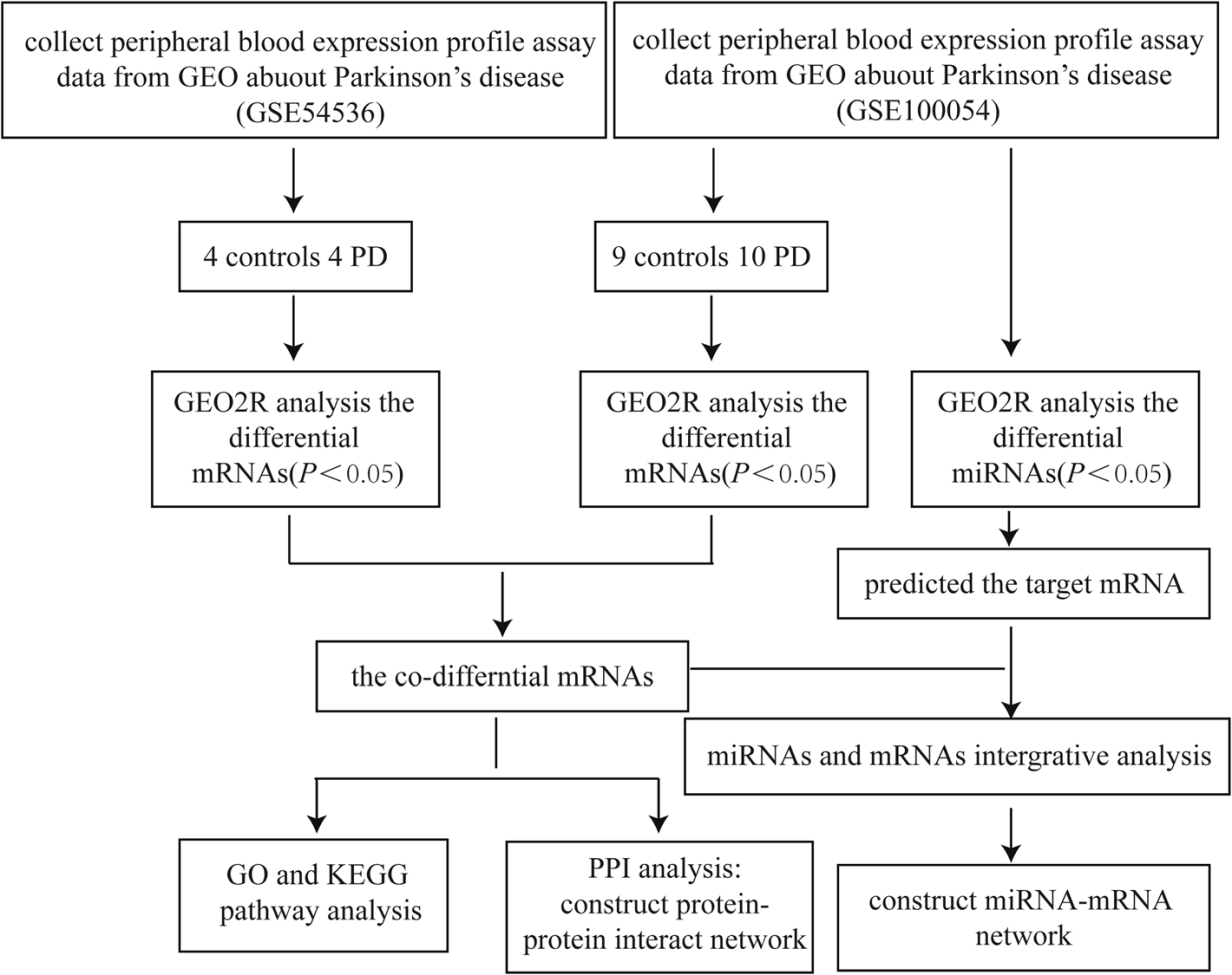
Flow chart of the data processing and analysis

We searched several key words, including “Parkinson’s Disease”, “blood”, “Expression profiling by array”, “*Homo sapiens*” and “20130101 to 20170531” in the GEO datasets. Two GSEs were selected in this study: GSE54536 and GSE100054. GSE54536 contains 4 controls and 4 untreated patients with stage 1 PD (Hoehn–Yahr scale). GSE100054 contains 9 controls and 10 PD patients. Figure 1 depicts the workflow of the data processing and analysis.

### Differentially expressed mRNAs and miRNAs selection

GEO2R (http://www.ncbi.nlm.nih.gov/geo/geo2r/) is an online analysis tool used to perform comparisons between groups of samples in a GEO dataset to identify DEGs or DEMs across experimental conditions. In our study, we obtained DEGs from two datasets, including GSE54536 and GSE100054. When we selected the DEGs, the cut-off criteria were *P*<0.05 and fold change ≥1.5. We analyzed the DEMs in the GSE100054 according to the cut-off criterion *P*<0.05 and fold change ≥2. The dataset of GSE54536 was analyzed with Illumina HumanHT-12 V4.0 expression beadchip, and GSE100054 was analyzed with Affymetrix Human Clariom D Assay.

### Function enrichment analysis and KEGG pathway analysis

The co-differential gene functional features in GSE54536 and GSE100054 related to PD were analyzed with DAVID [12]. DAVID is a web-based system that incorporates information from different resources to detect biological themes within the list of candidate genes. This includes evaluating the enrichment significance of gene ontology (GO) terms, and an analysis of the Kyoto Encyclopedia of Genes and Genomes (KEGG) pathways. Biological process, cellular components, and molecular function are included in GO terms. We defined a *P* value of less than 0.05 as significantly enriched.

### Protein-protein interaction (PPI) network construction analysis and identification of hub genes

The co-differential genes in the two datasets, GSE54536 and GSE100054, were used to construct the protein-protein interaction (PPI) network using the Search Tool for the Retrieval of Interacting Genes/Proteins (STRING, https://string-db.org/). We established the PPI network using only overlapped differentially expressed genes, and set confidence score cutoffs as greater than 0.4. Next, Molecular Complex Detection (MCODE) in Cytoscape was applied to determine the hub genes. Degree cutoff = 2, node score cutoff = 0.2, and K-core =2, and max. Depth = 100.

### Prediction of miRNA targets and construction of the miRNA-mRNA network

The DEMs in GSE100054 were used to predict the target genes using Targetscan (http://www.targetscan.org/). Targetscan is an online system that can predict target mRNA according to the miRNA seed region seeking the complementary specific sequence. In this study, we determined the intersection between the predicted miRNA target genes and the co-differentially expressed genes of GSE54536 and GSE100054. We then used these miRNA-mRNA regulatory networks and Cytoscape to visualize interactions between miRNAs and their potential targets in PD.

## Results

### Identification of DEGs and DEMs

**Fig. 2:**
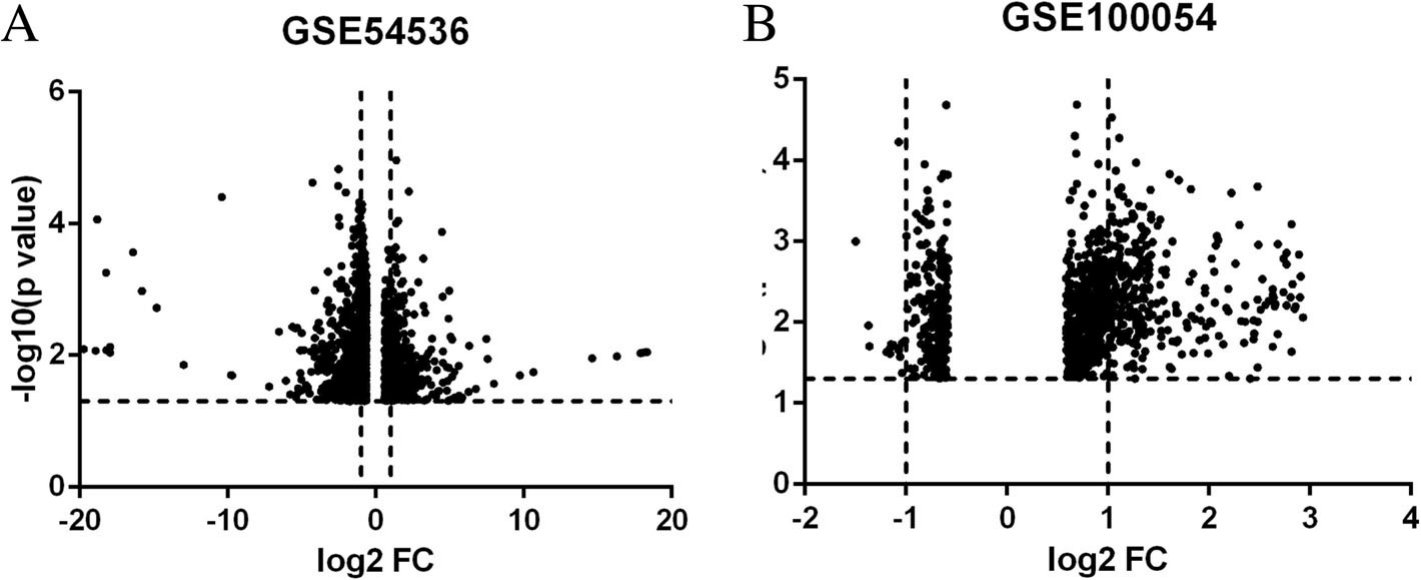
Volcano plot show the DEGs in GSE54536 and GSE100054

To detect the DEGs in GSE54536 and GSE100054, we set a cutoff of *P*<0.05 and a fold change of ≥1.5, and for the DEMs in GSE100054, *P*<0.05 and a fold change of 2. GEO2R analysis indicated the presence of 1790 and 967 DEGs in GSE54536 and GSE100054 respectively (Fig. 2a and b). In GSE54536 and GSE100054, there are 125 genes that co-exist in the DEGs. Among them, 17 genes are upregulated and 14 genes are downregulated compared to controls. We then screened out 42 DEMs from GSE100054 using the GEO2R analysis system.

### Functional enrichment and KEGG pathway analysis

**Fig. 3:**
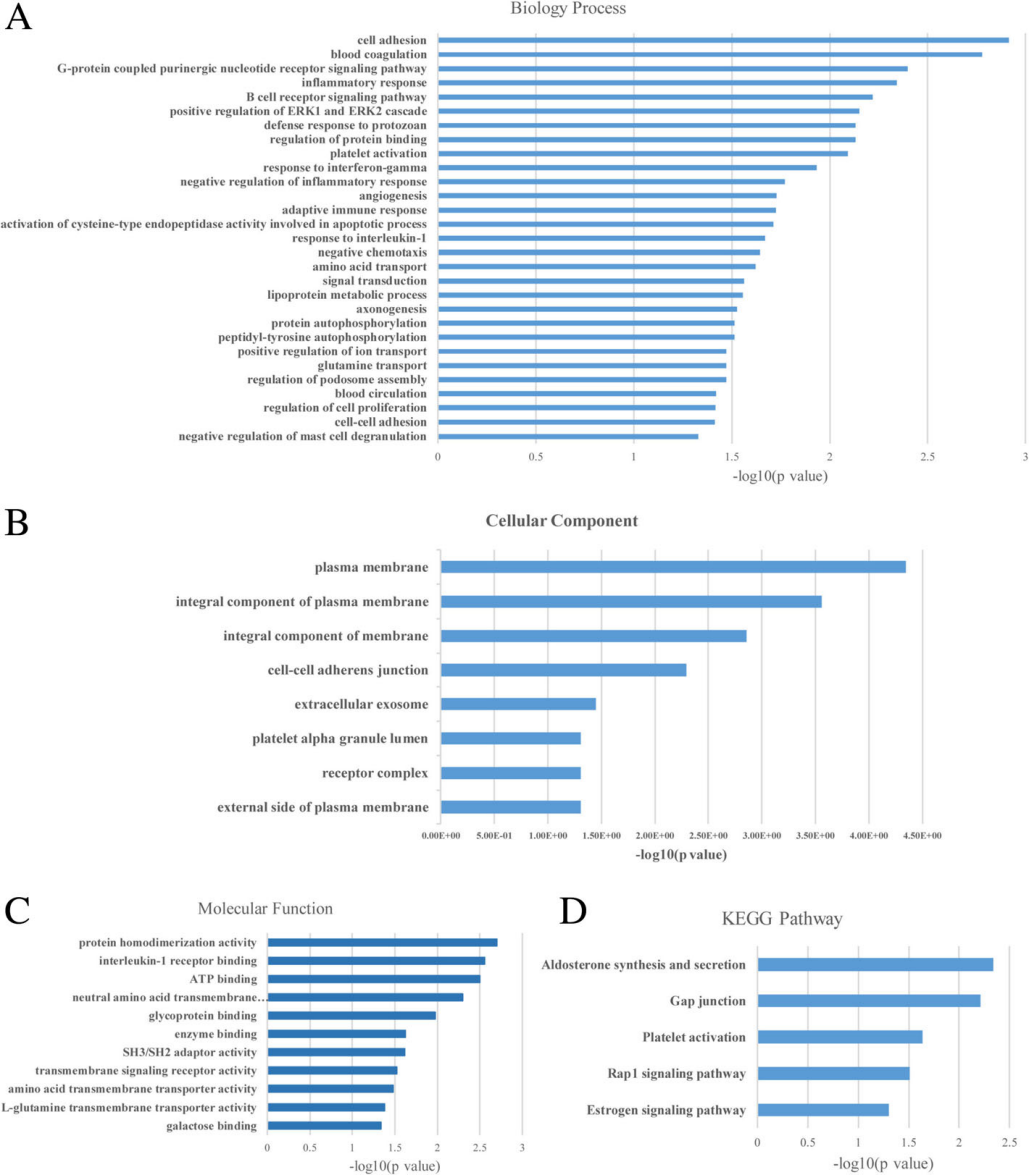
Enriched Gene Ontology terms of differentially expressed genes obtained from the Database for Annotation **a** biological process (BP); **b** cellular component (CC); **c** molecular function (MF). **d** enriched KEGG pathway

For the DEGs, the most significantly enriched GO terms on biological processes (BP) are Cell adhesion, Blood coagulation, G-protein coupled purinergic nucleotide receptor signaling, Inflammatory response, and B cell receptor signaling (Fig. 3a). We listed all the BP, cellular components (CC) (Fig. 3b), molecular functions (MF) (Fig. 3c), and KEGG pathways according to the GO terms (*P* < 0.05). In addition, the KEGG pathway analysis showed that DEGs were enriched in Aldosterone synthesis and secretion, Gap junctions, Platelet activation, Rap1 signaling, and Estrogen signaling (Fig. 3d).

### PPI network and identification of hub genes

**Fig. 4:**
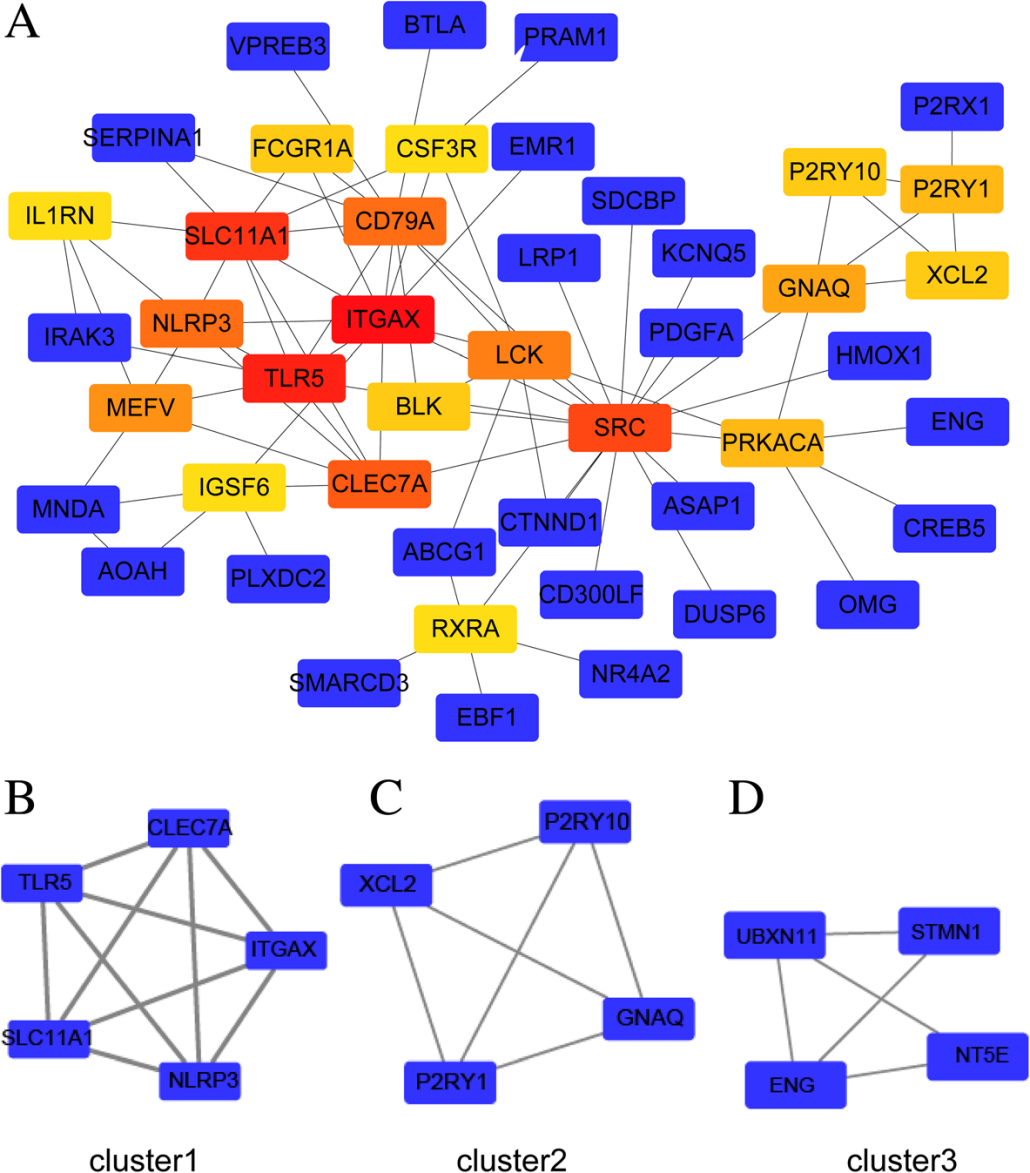
**a** show the protein-protein interaction network of DEGs in GSE54536 and GSE100054. The blue genes in **a** showed the common genes and from red to yellow show the top 20 hub genes in the network according to the criterion. **b**, **c** and **d** show the 3 clusters using MCODE

125 overlapping DEGs between GSE54536 and GSE100054 were used to establish the protein-protein (PPI) network by STRING, which composed 125 nodes and 93 edges. Subsequently, we analyzed the STRING results using CytoScape, and 20 genes in the PPI network were identified as hub genes in PD. Among others, these included ITGAX, TLR5, SLC11A1, SRC, CLEC7A, CD79A, LCK, and NLRP3 (Fig. 4a). Degree cutoff, node score cutoff, and k-core were set to 2, and max depth was set to 100.

We selected 3 clusters from the PPI network using MCODE, with the most significant cluster consisting of 5 nodes and 10 edges. MCODE analysis also showed that each cluster contained one “seed” gene. NRLRP3, XCL2, and NT5E are the seed genes in the three clusters respectively (Fig. 4b, c and d). The BP of hub genes were involved in Platelet activation, Inflammatory response, Innate immune response, B cell receptor signaling, Stimulatory C-type lectin receptor signaling, Lipopolysaccharide response, Leukocyte migration, and Regulation of cell proliferation (*P*<0.01).

### Construction of a miRNA target regulatory network

**Fig. 5:**
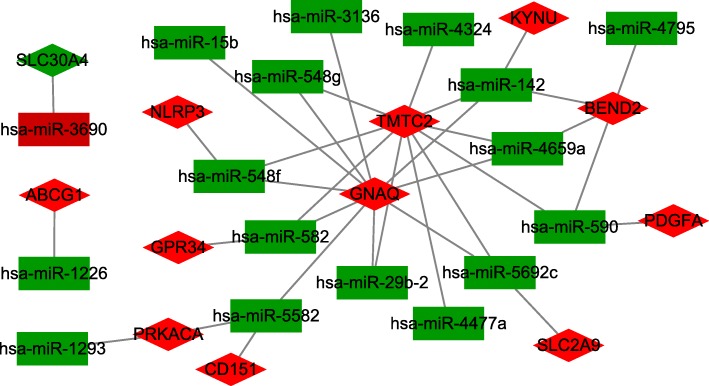
miRNA-mRNA regulation network of Parkinson’s disease. Diamond nodes represent target genes and rectangle nodes represent miRNAs. Green rectangles represent downregulation miRNAs in GSE100054, red rectangle represent upregulation miRNA. Green diamonds show the co-downregulated mRNA in GSE54536 and GSE100054, red diamonds show the co-upregulated mRNA in these two datasets

In the GSE100054 dataset, there are 42 differentially expressed pre-miRNAs. We examined the mature expression of these miRNA using miRBase. All mature miRNAs were uploaded to DIANA TOOLS (http://diana.imis.athena-innovation.gr/DianaTools/index.php?r=microT_CDS/index) to predict the targets of each. The overlapping mRNAs between the DIANA TOOLS predictions and the DEGs in GSE54536 and GSE100054 were then used to construct the regulatory network. After determining the intersection between the 125 DEGs and the predicted target genes of the DEMs, the miRNAs with no target genes or a negative regulatory relationship were discarded. That left 12 mRNAs and 17 miRNAs remaining, making up 34 miRNA-mRNA pairs. The relationships between mRNAs and miRNAs is shown in Fig. 5. Diamond-shaped nodes represent target genes, and rectangular nodes represent miRNAs. Green rectangles represent downregulation of miRNAs in GSE100054, while red nodes represent upregulation miRNAs. Green diamonds show the co-downregulated mRNAs in GSE54536 and GSE100054, and red diamonds show the co-upregulated mRNAs in these two datasets.

Among the 34 miRNA-mRNA pairs, only one miRNA was found to be increased in PD peripheral blood, while the other miRNAs were found to be reduced. Hsa-miR-3690 was elevated, and has a negative regulatory relationship with SLC30A4. We used Cytoscape APP centiscape 2.2 to analyze the network, and found that GNAQ and TMTC2 played important roles (centiscape2.2 degree = 10), and has-miR-142 was the most significant (centiscape2.2 degree = 4). GNAQ was negatively regulated by multiple miRNAs, including hsa-miR-142, hsa-miR-15b, hsa-miR-29b-2, hsa-miR-3136, hsa-miR-4659a, hsa-miR-548f, hsa-miR-548 g, hsa-miR-5582, hsa-miR-5692c, and hsa-miR-582. TMTC2 was negatively regulated by multiple miRNAs including hsa-miR-142, hsa-miR-29b-2, hsa-miR-4324, hsa-miR-4477a, hsa-miR-4659a, hsa-miR-548f, hsa-miR-548 g, hsa-miR-5692c, hsa-miR-582, and hsa-miR-590. Finally, BEND2 was negatively regulated by miRNAs including hsa-miR-142, hsa-miR-4659a, hsa-miR-4795, and hsa-miR-590. Given these observations, hsa-miR-142 is the most versatile and significant miRNA regulator in the network, and can impact GNAQ, TMTC2, BEND2 and KYNU (Fig. 5).

## Discussion

PD is a serious neurodegenerative disease. Onset and symptoms are influenced by genetic background, chronic inflammation, environmental toxicity, oxidative stress, mitochondria dysregulation, and calcium overload. Patients are often diagnosed with Parkinson’s disease via their motor symptoms, however this is far beyond the early stages of the disease. Prior to diagnosis, many people express no specific dyskinesia and instead show non-motor symptoms including mood and sleep disorders, visual impairment, fatigue, and depression [13, 14]. However, these nonspecific manifestations are rarely associated with Parkinson’s disease. Therefore, it is difficult to diagnose early-stage Parkinson’s disease. Our data suggest that Parkinson’s disease is a disease caused by multiple factors within multiple complex systems, with abundant crossovers between signaling pathways. For each patient, a comprehensive analysis focusing on biological function and interactions of PD-related genes can provide us valuable information to understand the pathogenesis of their individual disease. Our high throughout technique provides us with a powerful tool to analyze general characteristics and search for novel preventive and therapeutic interventions.

In this study, a total of 125 DEGs were found in GSE54536 and GSE100054, including 17 upregulated genes and 14 downregulated genes. All the DEGs were enriched in Cell adhesion, Blood coagulation, G-protein coupled purinergic nucleotide receptor signaling, Inflammatory response, and B cell receptor signaling. 20 genes were identified as hub genes in PD, including ITGAX, TLR5, SLC11A1, SRC, CLEC7A, CD79A, LCK, and NLRP3, due to their high degrees within the PPI network.

The results of this paper demonstrate that 17 miRNAs and 12 mRNAs form the regulatory network. Studies have confirmed that genes modulated during the pathogenesis of Parkinson’s disease appear in our regulatory network, and include CD79A [15] and NLRP3 [16, 17] in addition to miRNAs associated with PD models, including miR-142-3p [18], miR-15b-5p [19–21], and miR-590-3p [22] (Table 1).

**Table 1:** miRNAs/genes confirmed related to PD

miRNAs/genes	Disease	Variation tendency	Reference
CD79A	PD	down-regulated	Kobo H [15],
NLRP3	PD (mice Rotenone Exposure)	up-reglulated	Martinez EM [16], Zhou Y [17]
miR-142-3p	PD(patients plasma)	down-regulated	Chen L [18]
miR-15b-5p	PD(MPP^+^ damaged SH-SY5Y cells)	down-regulated	Xie N [19, 20]
miR-590-3p	(MPP^+^)-treated MES23.5 and SH-SY5Y cells	down-regulated	Wang J [22]

Here, a thorough analysis of the regulatory network revealed an extremely high degree of hsa-miR-142, hsa-miR-582, hsa-miR-590, hsa-miR-4659a, hsa-miR-548f, hsa-miR-548 g, hsa-miR-5582, GNAQ, TMTC2, and BEND2, indicating that these miRNAs and mRNAs may play key roles in the development of PD. miR-142 is highly correlated with inflammatory and immune responses in different physiological and disease [23]. There are two mature forms of miR-142: miR-142-3p and miR-142-5p. miR-142 is abundant in cells of hematopoietic lineage, and plays an important role in their differentiation [24, 25]. More importantly, miR-142 appears to have a strong effect on immune responses in a variety of physiological and disease states [26–29]. This is likely due to the fact that miR-142 targets genes belonging to the immune response pathway, and therefore can have a wide range of effects on multiple downstream signaling pathways. A recent study focused on plasma miRNAs, and showed that miR-142-3p was down regulated in PD patients compared with healthy controls [18]. Hsa-miR-582 function was involved in the regulation of cell proliferation, cell cycle progression, and apoptosis [30, 31]. hsa-miR-590 can negatively regulate the inflammatory response, chemokine secretion [32], and reactive oxygen biosynthesis [33]. However, the functions of hsa-miR-4659a, hsa-miR-548f, hsa-miR-548 g, and hsa-miR-5582 are not clear. G Protein Subunit Alpha Q (GNAQ) is involved in modulating or transducing various transmembrane signaling systems related to PD and ALS. It also can regulate B-cell selection and survival, and is required to prevent B-cell-dependent autoimmunity. TMTC2 is an integral membrane protein localized in the endoplasmic reticulum (ER). This protein plays a role in calcium homeostasis in the ER [34]. BEN Domain Containing 2 (BEND2) encodes a protein that participates in protein and DNA interactions during chromatin restructuring and transcription. These three genes (GNAQ, TMTC2, BEND2) were negatively regulated by miR-142 within the miRNA-mRNA network. These novel miRNAs and mRNAs, not previously confirmed to change in PD patients, may be new targets for intervention.

Our results suggest that miRNA-142 may be used as a potential biomarker. It may affect PD development via interaction with GNAQ, TMTC2, and BEND2. However, there is very little know about the relationship between specific genes and PD. While our results must be validated in vitro and in vivo, we believe that the genes we’ve identified could be potential targets for the diagnosis and treatment of PD. This study provides important information for the elucidation of the physiological and pathological processes governing PD.

## Abbreviations

BP: Biological process
CC: Cellular component
DEGs: the differentially expressed genes
DEMs: the differentially expressed miRNAs
ER: endoplasmic reticulum
GO: gene ontology
KEGG: Kyoto Encyclopedia of Genes and Genomes
MF: Molecular Function
miRNA: microRNA
PD: Parkinson’s disease
PPI: protein-protein interaction
RBD: rapid eye movement sleep disturbances
SN: Substantia Nigra

## References

[1] Kalia LV, Lang AE (2015). Parkinson's disease. Lancet.

[2] Wirdefeldt K, Adami HO, Cole P, Trichopoulos D, Mandel J (2011). Epidemiology and etiology of Parkinson's disease: a review of the evidence. Eur J Epidemiol.

[3] Dexter DT, Jenner P (2013). Parkinson disease: from pathology to molecular disease mechanisms. Free Radic Biol Med.

[4] Jankovic J (2008). Parkinson's disease: clinical features and diagnosis. J Neurol Neurosurg Psychiatry.

[5] Iranzo A, Santamaria J, Tolosa E (2016). Idiopathic rapid eye movement sleep behaviour disorder: diagnosis, management, and the need for neuroprotective interventions. Lancet Neurol.

[6] Tan CL, Plotkin JL, Venø MT (2013). MicroRNA-128 governs neuronal excitability and motor behavior in mice. Science.

[7] Pedersen ME, Snieckute G, Kagias K (2013). An epidermal microRNA regulates neuronal migration through control of the cellular glycosylation state. Science.

[8] Gomez GG, Volinia S, Croce CM (2014). Suppression of microRNA-9 by mutant EGFR signaling upregulates FOXP1 to enhance glioblastoma tumorigenicity. Cancer Res.

[9] Buchsbaum DJ, Croce CM (2014). Will detection of microRNA biomarkers in blood improve the diagnosis and survival of patients with pancreatic cancer. JAMA.

[10] Ben-Dov IZ, Tan YC, Morozov P (2014). Urine microRNA as potential biomarkers of autosomal dominant polycystic kidney disease progression: description of miRNA profiles at baseline. PLoS One.

[11] Kim J, Zhang Y, Skalski M (2014). microRNA-148a is a prognostic oncomiR that targets MIG6 and BIM to regulate EGFR and apoptosis in glioblastoma. Cancer Res.

[12] Huang dW, Sherman BT, Lempicki RA (2009). Systematic and integrative analysis of large gene lists using DAVID bioinformatics resources. Nat Protoc.

[13] Garcia-Ruiz PJ, Chaudhuri KR, Martinez-Martin P (2014). Non-motor symptoms of Parkinson's disease a review…from the past. J Neurol Sci.

[14] Rana AQ, Ahmed US, Chaudry ZM, Vasan S (2015). Parkinson's disease: a review of non-motor symptoms. Expert Rev Neurother.

[15] Kobo H, Bar-Shira A, Dahary D (2016). Down-regulation of B cell-related genes in peripheral blood leukocytes of Parkinson's disease patients with and without GBA mutations. Mol Genet Metab.

[16] Martinez EM, Young AL, Patankar YR (2017). Editor's highlight: Nlrp3 is required for inflammatory changes and Nigral cell loss resulting from chronic Intragastric rotenone exposure in mice. Toxicol Sci.

[17] Zhou Y, Lu M, Du RH (2016). MicroRNA-7 targets nod-like receptor protein 3 inflammasome to modulate neuroinflammation in the pathogenesis of Parkinson's disease. Mol Neurodegener.

[18] Chen L, Yang J, Lü J, Cao S, Zhao Q, Yu Z (2018). Identification of aberrant circulating miRNAs in Parkinson's disease plasma samples. Brain Behav.

[19] Xie N, Qi J, Li S, Deng J, Chen Y, Lian Y. Upregulated lncRNA small nucleolar RNA host gene 1 promotes 1-methyl-4-phenylpyridinium ion-induced cytotoxicity and reactive oxygen species production through miR-15b-5p/GSK3β axis in human dopaminergic SH-SY5Y cells. J Cell Biochem. 2018:1–12. 10.1002/jcb.27865 30302821

[20] Chen Y, Lian YJ, Ma YQ, Wu CJ, Zheng YK, Xie NC (2018). LncRNA SNHG1 promotes α-synuclein aggregation and toxicity by targeting miR-15b-5p to activate SIAH1 in human neuroblastoma SH-SY5Y cells. Neurotoxicology.

[21] Ding H, Huang Z, Chen M (2016). Identification of a panel of five serum miRNAs as a biomarker for Parkinson's disease. Parkinsonism Relat Disord.

[22] Wang J, Le T, Wei R, Jiao Y (2016). Knockdown of JMJD1C, a target gene of hsa-miR-590-3p, inhibits mitochondrial dysfunction and oxidative stress in MPP+−treated MES23.5 and SH-SY5Y cells. Cell Mol Biol (Noisy-le-grand).

[23] Sharma S (2017). Immunomodulation: a definitive role of microRNA-142. Dev Comp Immunol.

[24] Dahlhaus M, Roolf C, Ruck S, Lange S, Freund M, Junghanss C (2013). Expression and prognostic significance of hsa-miR-142-3p in acute leukemias. Neoplasma.

[25] Lu X, Li X, He Q (2013). miR-142-3p regulates the formation and differentiation of hematopoietic stem cells in vertebrates. Cell Res.

[26] Kumar P, Dezso Z, MacKenzie C (2013). Circulating miRNA biomarkers for Alzheimer's disease. PLoS One.

[27] Makino K, Jinnin M, Kajihara I (2012). Circulating miR-142-3p levels in patients with systemic sclerosis. Clin Exp Dermatol.

[28] Maes T, Cobos FA, Schleich F (2016). Asthma inflammatory phenotypes show differential microRNA expression in sputum. J Allergy Clin Immunol.

[29] Mandolesi G, De Vito F, Musella A (2017). miR-142-3p is a key regulator of IL-1β-dependent Synaptopathy in Neuroinflammation. J Neurosci.

[30] Zhang X, Zhang Y, Yang J, Li S, Chen J (2015). Upregulation of miR-582-5p inhibits cell proliferation, cell cycle progression and invasion by targeting Rab27a in human colorectal carcinoma. Cancer Gene Ther.

[31] Liu Y, Jiang J, Wang X, Zhai F, Cheng X (2013). miR-582-5p is upregulated in patients with active tuberculosis and inhibits apoptosis of monocytes by targeting FOXO1. PLoS One.

[32] He PP, Ouyang XP, Tang YY (2014). MicroRNA-590 attenuates lipid accumulation and pro-inflammatory cytokine secretion by targeting lipoprotein lipase gene in human THP-1 macrophages. Biochimie.

[33] Bao MH, Li JM, Zhou QL (2016). Effects of miR-590 on oxLDL-induced endothelial cell apoptosis: roles of p53 and NF-κB. Mol Med Rep.

[34] Sunryd JC, Cheon B, Graham JB, Giorda KM, Fissore RA, Hebert DN (2014). TMTC1 and TMTC2 are novel endoplasmic reticulum tetratricopeptide repeat-containing adapter proteins involved in calcium homeostasis. J Biol Chem.

