# Correction to: miRNAs and target genes in the blood as biomarkers for the early diagnosis of Parkinson’s disease

**DOI:** 10.1186/s12918-019-0701-3

**Published:** 2019-02-12

**Authors:** Xiaoting Liu, Jinhu Chen, Tianyuan Guan, Hui Yao, Wenpei Zhang, Zhenlong Guan, Yanqin Wang

**Affiliations:** 1 Department of Physiology, College of Life Science, Hebei Normal University, Shijiazhuang, China; 2 Department of Endocrinology, Hebei General Hospital, Shijiazhuang, China; 3 Department of Neurology, Hebei General Hospital, Shijiazhuang, China

**Correction to: BMC Syst Biol (2019) 13:10**

**https://doi.org/10.1186/s12918-019-0680-4**

It was highlighted that the original article [1] contained some typesetting mistakes in the first paragraph of the Background section. This Correction article shows the incorrect and correct first paragraph. The original article has been updated.

**Incorrect**

Parkinson’s disease (PD) is the second most common neurodegenerative disease behind Alzheimer’s disease. It has a 1% incidence in people 65 years of age [1], and 4% in those 80 years of age [2]. Early stage PD patients showed only non-motor symptoms (NMS), such as constipation, olfactory dysfunction, depression, and sleep disorders [3]. Clinical motor symptoms included bradykinesia, stiffness, tremor and postural instability, and asymmetric seizures. Other motor dysfunctions included gait and posture changes (manifesting as panic gait or walking forward chaotically with rapid bending), speech and swallowing difficulties, and masked expressions [4]. When people displayed clinical symptoms, there was an associated loss of at least 50% of dopaminergic neurons in the substantia nigra (SN), and an 80% decrease in striatal DA content. Early identification of PD molecular biomarkers is critical for initiating timely treatment prior to the onset of motor symptoms. This is especially true given that 90% of rapid eye movement sleep behavior disorder (RBD) patients develop PD in most cases, Do non-motor symptoms need change to NMS? precede motor symptoms [5]. However, these Do non-motor symptoms need change to NMS? are easily overlooked. Therefore, the early recognition and treatment of Do non-motor symptoms need change to NMS? is not only one of the most important and difficult issues in the current diagnosis and treatment of PD, but also directly affects the overall therapeutic effect on PD patients. Currently, the pathogenesis of PD is not clear. PD is caused by a variety of factors, including age, genetics, oxidative stress, calcium overload, mitochondrial dysfunction, and environmental factors. Previous studies have focused on individual pathogenic factors, but PD is not caused by a single factor, and there is cross-talk among many of the causal elements. These factors form a regulatory network, and we should re-examine Do Parkinson’s disease need change to PD? adopting a network perspective to better comprehend the complex interplay of causes. Due to the scarcity of brain tissue, it is necessary to establish a database of clinical data of PD patients and bioinformatic libraries to explore incidence, related risk factors, biomarkers, occurrence, and development.

**Correct**

Parkinson’s disease (PD) is the second most common neurodegenerative disease behind Alzheimer’s disease. It has a 1% incidence in people 65 years of age [1], and 4% in those 80 years of age [2]. Early stage PD patients showed only non-motor symptoms (NMS), such as constipation, olfactory dysfunction, depression, and sleep disorders [3]. Clinical motor symptoms included bradykinesia, stiffness, tremor and postural instability, and asymmetric seizures. Other motor dysfunctions included gait and posture changes (manifesting as panic gait or walking forward chaotically with rapid bending), speech and swallowing difficulties, and masked expressions [4]. When people displayed clinical symptoms, there was an associated loss of at least 50% of dopaminergic neurons in the substantia nigra (SN), and an 80% decrease in striatal DA content. Early identification of PD molecular biomarkers is critical for initiating timely treatment prior to the onset of motor symptoms. This is especially true given that 90% of rapid eye movement sleep behavior disorder (RBD) patients develop PD in most cases, NMS precede motor symptoms [5]. However, these NMS are easily overlooked. Therefore, the early recognition and treatment of NMS is not only one of the most important and difficult issues in the current diagnosis and treatment of PD, but also directly affects the overall therapeutic effect on PD patients. Currently, the pathogenesis of PD is not clear. PD is caused by a variety of factors, including age, genetics, oxidative stress, calcium overload, mitochondrial dysfunction, and environmental factors. Previous studies have focused on individual pathogenic factors, but PD is not caused by a single factor, and there is cross-talk among many of the causal elements. These factors form a regulatory network, and we should re-examine PD adopting a network perspective to better comprehend the complex interplay of causes. Due to the scarcity of brain tissue, it is necessary to establish a database of clinical data of PD patients and bioinformatic libraries to explore incidence, related risk factors, biomarkers, occurrence, and development.
